# Dislocation of the Outer End of the Clavicle

**Published:** 1903-11-07

**Authors:** 


### DISLOCATION OF THE OUTER END OF THE CLAVICLE.

This injury receives but scant notice in the majority of text-books of surgery, and yet it is of some importance to be quite clear, in dealing with any particular instance in which such a dislocation has occurred, what is the best line of treatment to adopt, and what prognosis can be given. For practical purposes Dr. John G. Sheldon[1] classifies all cases into two groups: (1) Cases in which the coraco-clavicular ligaments are completely ruptured, and (2) cases in which the coraco-clavicular ligaments are not completely ruptured. He believes that it is possble by physical examination to distinguish these two classes clinically; and the importance of the matter lies in this, that if the coraco-clavicular ligaments are torn through operative measures will be required to secure a satisfactory result, whereas when the ligaments are not quite torn through, a cutting operation will not, as a rule, be called for. The signs which Dr. Sheldon considers may be depended upon to indicate rupture of the coraco-clavicular ligaments are (1) inability to reduce the dislocation; (2) separation of the articular surfaces more than one inch; (3) ability to produce a longitudinal separation of the articular surfaces one-third of an inch, or to easily elevate the outer end of the clavicle; and (4) marked tendency to recurrence of the dislocation. Although cases have been reported which showed these symptoms were treated by non-operative measures and resulted in no impairment of function, yet the deformity which nearly always remains is in itself objectionable, and with regard to function this is not always completely regained, and has in some instances been so much impaired that the patient has been unable to raise the arm from the side. With the aid of an operation a good result may be safely predicted. The best method is to expose the joint by incision, drill the clavicle and acromion and fasten them together with absorbable sutures, approximate the edges of torn ligaments and fascia with fine catgut, and suture the skin incision. Dr. Sheldon prefers to use absorbable sutures because they are as effective as wire, while if the latter be used it is liable to become a source of irritation and its removal may become necessary.

[1] Annals of Surgery, September, 1903.

